# Magnitude and Spread of Bed Bugs (*Cimex lectularius*) throughout Ohio (USA) Revealed by Surveys of Pest Management Industry

**DOI:** 10.3390/insects12020133

**Published:** 2021-02-04

**Authors:** Susan C. Jones

**Affiliations:** Department of Entomology, The Ohio State University, Columbus, OH 43210-1065, USA; jones.1800@osu.edu

**Keywords:** *Cimex lectularius*, integrated pest management, pesticide misuse, pest survey, urban areas

## Abstract

**Simple Summary:**

Bed bugs are small blood-sucking insects that live indoors and feed on humans. They have become a problem in countries worldwide. In this study, the problem in Ohio (Midwest U.S.) was measured based on treatments by licensed pest control companies throughout the state. Results from 2005 showed that Ohio’s bed bug problem likely started in Hamilton County, which includes Cincinnati. Much larger numbers of bed bug treatments were performed in 2011 and again in 2016, especially in counties with large cities. Almost every Ohio county had numerous bed bug treatments in 2016. Most treatments were in apartments/condos and single-family homes. Residents misused many pesticides, especially over-the-counter “bug bombs” and household cleaners, trying to eliminate bed bugs. Many people also threw away unwrapped infested furniture, which may further spread these bugs. More public education is needed to stop such practices. This study shows that bed bug problems can grow and spread quickly. Federal, state, and local officials and the public should immediately deal with bed bugs rather than waiting until they become an even bigger problem.

**Abstract:**

Bed bugs have recently re-emerged as human pests worldwide. In this study, two surveys queried licensed pest management companies in Ohio (Midwest USA) about their experiences managing bed bugs. A primary objective was to assess the magnitude and spread of bed bug infestations statewide based on companies’ treatment records from 2005 and 2011 (first survey) and 2016 (second survey). The survey response rates were 35.6% and 31.6%, respectively. Treatment data from 2005 indicated that Ohio’s bed bug problem likely started in the SW corner of the state in Hamilton County (includes city of Cincinnati), since it totaled five times more treatments (approximately 4500) than second-ranking, centrally located Franklin County (Columbus). In the first half of 2011, more than 15,000 treatments were performed in these two counties. In 2016, treatments reached nearly 38,000 in Franklin County and in NE Ohio in the three combined counties that include Cleveland-Akron-Canton. Bed bug problems expanded statewide during an 11 y period, with an estimated 100+ treatments in 7 counties in 2005, 45 counties in 2011, and nearly all 88 counties in 2016. Apartments/condos and single-family residences comprised the largest share of bed bug work. Residents misused many pesticides and household cleaners trying to eliminate bed bugs. Many also discarded unwrapped infested furniture, which may further spread these bugs. More public education is needed to stop such practices. This study shows that bed bug problems grow and spread quickly; federal, state, and local officials and the public should immediately deal with bed bugs.

## 1. Introduction

In the last two decades, bed bugs have resurged in industrialized regions worldwide, particularly in North America [1,2,3,4,5,6,7,8], Europe [9,10], and Australia [11]. The United States is among the counties that have been severely impacted by bed bugs. The species found throughout the U.S. is *Cimex lectularius*, which is known as the common bed bug or simply the bed bug.

Insights into the biology and behavior of bed bugs [12,13,14,15,16] help shed light on how these small, blood-sucking insects quickly become major problems in diverse settings such as private residences, apartments, hotels, nursing homes, day care centers, hospitals, and office buildings. Bed bugs live indoors and preferentially feed on humans. They are nocturnal and therefore most active at night. Bed bugs often hide near human resting and sleeping places, but their flat shape and climbing ability enable them to hide in narrow cracks and crevices anywhere from floor to ceiling. Research has shown that approximately 80% of bed bugs remain hidden, with only hungry bugs venturing from their hiding places [17]. Bed bugs have a fast life cycle, going from egg to reproductive adult within several weeks or longer depending on temperature. As the number of bed bugs increases in a home, the bugs tend to become more and more widely dispersed. These bugs can walk long distances indoors, but they also hitchhike on items and are easily transported to new locations [17,18,19].

Bed bugs are difficult and expensive to control, and they necessitate multiple integrated pest management (IPM) strategies. If left untreated or improperly treated, bed bugs rapidly increase in number. Bed bugs adversely affect public health and well-being of all socioeconomic classes, but particularly those who cannot afford the cost, time, and effort needed to control these pests [20,21]. There is a higher risk of bed bug infestations in poorer neighborhoods, in areas where evictions were more prevalent, and in more crowded neighborhoods [22].

Annual reports from two nationwide pest management companies, Orkin and Terminix, since 2010–2011 have consistently listed one or more Ohio cities among the most highly bed bug infested in the nation [23,24,25,26,27]. In 2010, Ohio was termed “the most bed bug-infested state,” because it had the most highly ranked cities on Terminix’s list (Cincinnati #4, Columbus #7, Dayton #8, Cleveland #14) [23]. Although the rankings by nationwide companies are skewed by their predominance or lack thereof in particular geographic areas, they indicate that bed bugs are a problem in Ohio.

**Figure 1:**
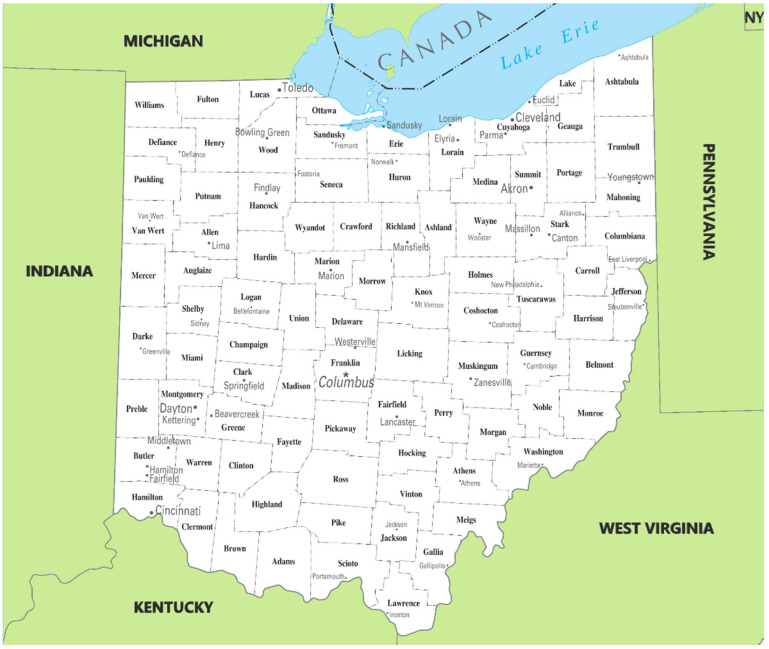
Map of Ohio depicting its 88 counties, major cities, and some towns (star indicates state capital, Columbus). Map modified from http://mapsof.net/ohio/ohio-cities-and-towns (accessed on 3 February 2021).

Ohio is in the Midwest U.S. and is the 7th most populous and 10th most densely populated of the 50 states [28]. Based on 2016 population estimates [28], the most populous of Ohio’s 88 counties (Figure 1) are: (1) Franklin County [state capital of Columbus] with 1.27 million people; (2) Cuyahoga County [Cleveland] with 1.25 million; (3) Hamilton County [Cincinnati] with 811,000; (4) Summit County [Akron] with 541,000; (5) Montgomery County [Dayton] with 532,000; (6) Lucas County [Toledo] with 433,000. These major cities are widely distributed throughout the state and are connected by a well-developed intrastate and interstate highway system, which also provides access to five bordering states and beyond. Eleven of Ohio’s metropolitan areas are within a day’s drive of approximately 50% of the U.S. population [29].

Despite the bed bug resurgence in the U.S., few long-term data have been compiled to trace the timeline and scope of bed bug infestations on a statewide basis. A primary objective of the current study was to assess the magnitude and spread of bed bug infestations throughout Ohio based on licensed companies’ treatment records spanning an 11 y period (2005 through 2016). A secondary objective was to obtain information on residents’ responses to bed bugs, including misidentification, do-it-yourself (DIY) pest control, and furniture disposal.

## 2. Materials and Methods

Two statewide surveys of Ohio’s licensed pest management professionals (PMPs) were conducted asking about their experiences with bed bugs. Both questionnaires covered topics such as bed bug infestation frequency, detection and treatment practices, and products used by PMPs and the public. The first survey requested data from the first half of 2011, but it also asked for treatment numbers from 2005 when available. The second survey requested data from 2016.

Survey questions and the statistical plan were developed by the author in consultation with Ohio State University’s Statistical Consulting Service (SCS). Survey questions were reviewed by several members of the Ohio Pest Management Association (OPMA) prior to being finalized. The Ohio State University Institutional Review Board (IRB) reviewed and pre-approved both surveys for distribution—they were determined to be exempt from federal regulations governing the conduct of human subjects’ research.

The distribution list for both surveys was obtained through the Ohio Department of Agriculture (ODA) Pesticide and Fertilizer Regulation Division. All licensed commercial applicators in Category 10a, ‘General Pest Control,’ in Ohio in 2011 and 2016, respectively, were listed along with company name, mailing address, and county; an email address occasionally was provided. The list of licensees was refined to avoid sending a duplicate survey to the same company or franchise branch office; surveys were sent to each company owner or manager, who was identified using Internet searches or phone calls when unknown. Email addresses were obtained similarly.

SCS administered the surveys online or by U.S. mail. The first survey ran from 19 August to 30 September 2011. The second survey ran from 22 February to 21 March 2017. Both surveys consisted of 29 questions that were formatted as open-ended or fixed responses. Many questions were identical in both surveys. Exhibit S1: 2011 Ohio Bed Bug Survey Questionnaire; and Exhibit S2: 2016 Ohio Bed Bug Survey Questionnaire are provided in the Supplementary Materials.

### 2.1. Calculation of Total Number of Treatments Per County in 2005, 2011, and 2016

In both surveys, each company was asked to provide information on the total number of bed bug treatments per year using predefined categories or intervals: 0, 1–10, 11–50, 51–100, 101–500, 501–1000, 1001–2000, 2001–3000, and >3000. (The intervals are intentionally narrower for smaller treatment numbers since this allowed better assessment of growth of the bed bug problem.) The median value of these intervals was used to convert categorical variables into numerical variables. The number 3500 was used for >3000. ‘Did not provide treatment’ was considered as 0, and ‘Don’t know’ was treated as missing data. Further, each company was asked to provide information on the percentage of their overall bed bug work in each Ohio county in 2011 (first survey) and 2016 (second survey). Therefore, the number of bed bug treatments by each company in each county was calculated by multiplying the number of treatments (median) with the corresponding weight in the county category. Then the number of bed bug treatments in a county was summed for all companies working there based on survey data. For example, if 4 of 6 company respondents that worked in county X reported annual treatment numbers of 5.5 (median of 1–10), 30.5 (median of 11–50), 75.5 (median of 51–100), and 300.5 (median of 101–500), and their percentage of work in county X was 90%, 60%, 10%, and 10%, respectively, then the number of treatments in county X based on survey data was 61.3 [5.5(0.9) + 30.5(0.6) + 75.5(0.1) + 300.5(0.1)]. If all 4 companies worked exclusively in county X, the number of treatments would be 412 (5.5 + 30.5 + 75.5 + 300.5).

Additionally, information on the total population of licensed companies in each Ohio county was available in the ODA distribution lists from 2011 and 2016. Companies that did not perform bed bug work were removed from each list based on (1) survey responses and (2) a search of exclusionary key words, such as bird, lawn, landscape, tree, weed, and wildlife, in their company name or email. There were an estimated 475 companies in Ohio that provided bed bug treatments in 2011 (first survey) and 797 companies in 2016 (second survey). The number of bed bug treatments in a county (survey data) was adjusted by the response rate in that county (total number of companies in a county/the number of companies in county that responded to the survey). Continuing with the above example for county X, if 12 licensed companies were listed in that county during 2011, and a sum of 61 treatments was provided by 4 of 6 survey respondents working in that county, then 61(12/6) = 122.6 total treatments. In this way, the estimated total number of bed bug treatments in each county was determined for 2005, 2011, and 2016, and these numerical values were used to plot heat maps [30].

Note that 2005 estimates have the most uncertainty since adequate information was unavailable that year regarding the total population of companies per county and the percentage of work performed by each company in each county. Estimates of total treatments in 2005 are based on the total population of companies per county in 2011 and the percentage of work performed by each company in each county in 2011.

### 2.2. Treatment Sites and Treatment Types

Each company was asked to indicate sites (i.e., hotels, office buildings, hospitals, schools, vehicles, etc.) where they provided bed bug treatments and the percentage of their overall bed bug work at each site in 2011 and 2016. The number of treatments per site was calculated by multiplying the number of treatments per company with the corresponding weight in the site category, and then summing for all companies. Companies also were asked to provide information on the types of treatments that they used such as insecticides, structural fumigation, heat and cold treatment, and laundering.

### 2.3. Statistical Analyses

Basic summary statistics were calculated for survey responses. These statistics included the number of respondents to each question, the frequency of responses to questions where the respondent was given several choices, and the mean and standard deviation for questions where the required response was a number. The Chi-Square Test of Independence (alpha = 0.05) was used to determine whether categorical responses in 2011 and 2016 were independent or related. SPSS software (Statistical Product and Service Solutions; IBM Corp., Armonk, NY, USA) was used for data analyses.

## 3. Results and Discussion

### 3.1. Number of Survey Respondents and Company Characteristics

The response rate for the first survey, in 2011, was 35.6% (225/632). The mode of response was almost evenly split between mail (n = 123, 54.7%) and online (n = 102, 45.3%). Of the 202 respondents providing information on their business location, 91.6% were in Ohio (59 of 88 counties) and 8.4% were situated out of state but doing pest control in Ohio. The oldest companies were established in the early 1900s (n = 2). The largest numbers of companies were established in 2010 and 2011. The midpoint was 1994, with 50% of respondents established before and after that time. A total of 205 companies provided information on the number of employees during 2011—a few companies consisted of only the owner (n = 6, 2.9%); most companies had ≤5 employees (n = 145, 70.7%); and the largest company had 80 employees.

The response rate for the second survey, which requested data for 2016, was 31.6% (329/1041). In contrast to the first survey, the mode of response was primarily online (90.9%). Business location was provided by 309 respondents. Companies were widely distributed throughout the state, occurring in 65 of 88 counties. The oldest companies were established in the early 1900s (n = 3). There was a large spike in the number of companies in 2012 and 2013. The midpoint was in year 2000. A total of 256 companies provided information on their employee numbers during 2016—as in the first survey, a few companies consisted of only the owner (n = 6, 2.3%) and the majority of companies had ≤5 employees (n = 177, 69.1%). However, there were more larger companies in 2016 than in 2011, with 3.1% of respondents having 100 to 450 employees and one company having 2000 employees.

### 3.2. Bed Bug Treatments by Licensed PMPs

In the first survey, 208 companies answered the question “Do you provide treatment services for bed bugs?” with 140 (67.3%) answering “Yes” and 68 (32.7%) answering “No” for 2011. In the second survey, 309 companies answered this same question, with 204 (66.0%) answering affirmatively for 2016. This percentage is comparable to the first survey. Ohio’s bed bug data are based on the 140 companies that provided bed bug treatment services in 2011 and the 204 companies that did so in 2016.

Some general trends were evident regarding company location and bed bug treatments. Companies located in counties with large metropolitan areas tended to be more numerous and their bed bug work was concentrated in their home county. However, larger companies were most likely to perform bed bug work in multiple counties. Companies located in rural counties tended to be smaller and their bed bug work often was more dispersed, encompassing their home county and one or more adjacent counties.

#### 3.2.1. Estimated Total Numbers of Treatments and Geographic Distribution in Ohio

**Figure 2:**
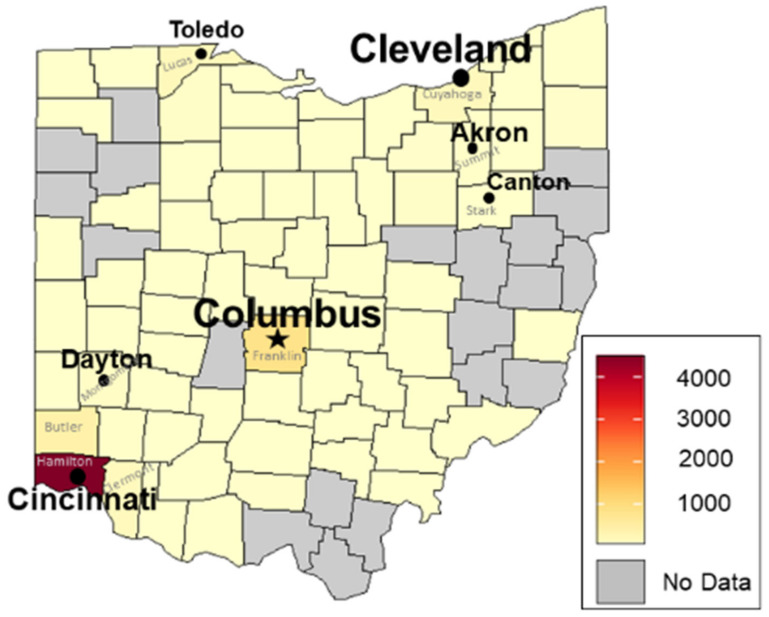
Heat map of estimated total number of bed bug treatments performed by licensed companies in each Ohio county during 2005. As shown in the key, the darkest shade of orange red indicates a maximum threshold of approximately 4500 treatments.

Treatment data strongly suggest that Ohio’s bed bug problem started in the extreme SW corner of the state in Hamilton County. In 2005, Hamilton County had five times more total treatments (approximately 4500) than the second-ranking county, centrally located Franklin County, with approximately 850 treatments (Figure 2). This occurred despite Franklin County being more populous. Additionally, there appeared to be spillover of the bed bug problem from Hamilton County into nearby counties (Figure 2): Butler County had approximately 450 treatments and Clermont County had approximately 200 treatments; Montgomery County had almost 100 treatments. In my capacity as Ohio State University’s State Extension Specialist on Household Pests, I received my first call about bed bugs in 2004 from Cincinnati—the caller, a senior citizen, described living in a government-subsidized high-rise building that was over-run with bed bugs. Additional evidence of the bed bug problem in Hamilton County in the early to mid-2000s is provided by Hamilton County Public Health, which recorded its initial bed bug complaints in 2003 and 2004 (2 complaints each year) followed by 37 complaints in 2005 [21]. In contrast, Columbus Code Enforcement received its first bed bug complaints (4) in 2005 (S. Carpenter, personal communication) [31].

Elsewhere in Ohio during 2005, more bed bug treatments were performed in counties with large cities (Figure 2): Lucas County (Toledo) and Cuyahoga County (Cleveland) had approximately 200 treatments each. Otherwise, most counties in Ohio (52) had approximately 15 or fewer total treatments, with 36 of these counties having 2 or fewer treatments. Note, however, that these lower numbers may not be very reliable since having too few points upon which to base the surface typically leads to larger errors in heat maps [30]. No data were provided for 20 counties. This is consistent with the relatively small number of companies (n = 62, 44.5% of respondents) that did bed bug work in 2005.

The approach for estimating total treatments in 2005 can lead to errors due to reliance on 2011 data regarding the total population of licensed companies and the percentage of each company’s work per county (see Materials and Methods). Reliance on these 2011 data likely led to underestimates of total treatments per county in 2005, since the total population of licensed companies should have been smaller in 2005 and bed bug jobs also should have been more concentrated in the relatively few counties with bed bugs (Figure 2).

Nonetheless, the raw data lend support for the abovementioned treatment estimates, with companies located in 19 counties reporting that they treated for bed bugs during 2005. Six of 14 company respondents in Hamilton County plus 3 of 4 out-of-state companies that did most work there reported median treatment numbers of 5.5, 750.5, 30.5, 5.5, 5.5, 750.5, 5.5(0.7), 3500(0.9), and 75.5(0.75), respectively. These data include the largest number of treatments during 2005, which was reported by a relatively large, well-established company (upper 15% of company sizes in Ohio and >30 years old in 2005). In nearby Montgomery County, 2 of 6 companies both reported 5.5 treatments during 2005. Low numbers of treatments also were reported in 7 additional counties in SW Ohio, with 5.5 treatments each in Brown, Greene, Highland, Preble, and Warren counties and 30.5 treatments each in Butler and Clermont counties. Hence the raw data from 2005 lend strong support for the hypothesis that Ohio’s bed bug problem originated in Hamilton County. Furthermore, compared to the 2005 heat map (Figure 2), these raw data more clearly demonstrate that the bed bug problem was becoming widespread in SW Ohio in 2005.

In central Ohio, raw data indicated that 10 of 20 company respondents in Franklin County, the most populous county in the state, reported bed bug treatments ranging from 5.5 to 300.5 during 2005. In the adjacent counties of Delaware and Licking, a single company from each county reported 5.5 bed bug treatments. In NW Ohio, 3 of 5 companies located in Lucas County reported from 5.5 to 75.5 treatments. In NE Ohio, Cuyahoga and Summit counties also had companies reporting low numbers of treatments (5.5 to 30.5). Similarly low treatment numbers were reported by companies located in the remaining 4 of 19 counties (Belmont, Hocking, Pike, Portage). Hence, the raw data and the 2005 heat map (Figure 2) both showed that bed bug treatments generally were more numerous in Ohio counties with large cities. Overall, the raw data support the conclusions drawn from the 2005 heat map.

**Figure 3:**
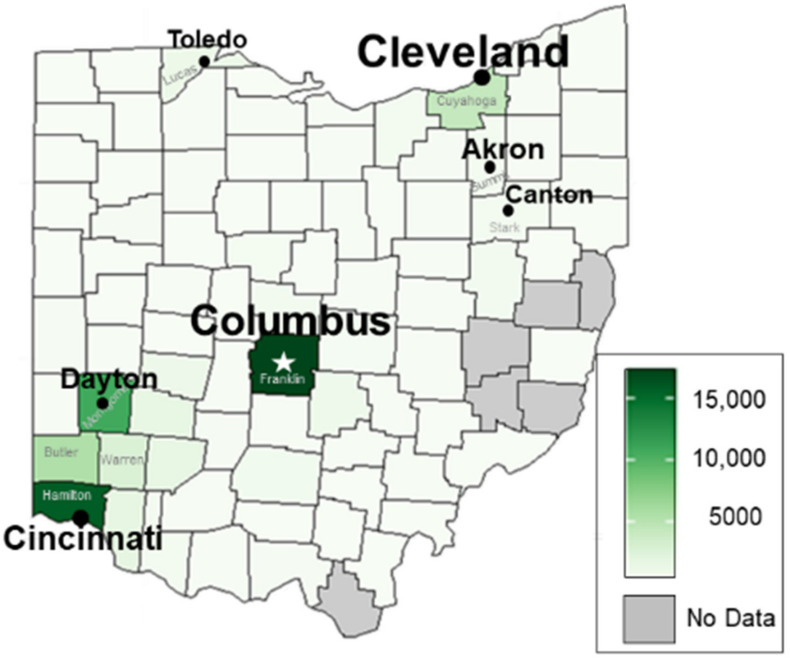
Heat map of estimated total number of bed bug treatments performed by licensed companies in each Ohio county during first half of 2011. The darkest shade of green indicates a maximum threshold of approximately 17,000 treatments.

The maximum number of treatments in Ohio counties greatly increased from 2005 (Figure 2) to mid-2011 (Figure 3). Since only 6 months of treatment data were obtained for 2011, it is likely that the following treatment numbers throughout the state would have increased, perhaps doubled, by the end of the year.

In mid-2011, Franklin and Hamilton counties still had the most bed bug treatments (Figure 3), with approximately 17,000 and 16,000, respectively. The growing bed bug problem in both counties is consistent with increasing complaints to city and county health departments. For example, the Cincinnati Health Department registered more than 700 complaints in 2007 (the first year that it compiled these data) and just over 1100 complaints in 2008 [21]. Columbus Code Enforcement recorded 33 complaints in 2007, with that number increasing to 178 the next year [31]. Furthermore, in 2008, growing concerns about bed bugs led to the formation of local bed bug task forces: The Joint Bed Bug Task Force in Cincinnati/Hamilton County and the Central Ohio Bed Bug Task Force in Columbus/Franklin County; I was a member from their inception onward. In early 2009, an informal survey of selected pest control companies revealed that numerous companies in Cincinnati received hundreds of bed bug calls weekly (M. Beal, personal communication) [32]. Cincinnati ranked #1 in the nation in both 2010 and 2011 based on the number of annual bed bug treatments performed by Orkin; Columbus ranked close behind at #3 in 2010 and #6 in 2011 [24].

In addition to Hamilton County, large numbers of treatments predominated in counties in the SW corner of the state (Figure 3), with Montgomery, Butler, and Warren counties estimated at 11,000; 5500; and 3000 total treatments, respectively. In 2009, the “Bed Bug War” in downtown Dayton was featured in The Dayton Daily News when a high-rise apartment building required fumigation because it was so heavily infested with bed bugs [33].

In NE Ohio during 2011, Cuyahoga County had approximately 5000 total treatments, with approximately 500 treatments in both Summit and Stark counties. In NW Ohio, Lucas County had approximately 1200 treatments.

Bed bug problems also appeared to have spread to many other counties by mid-2011, with an estimated 100 treatments or more in 45 of Ohio’s 88 counties. Approximately 15 or fewer treatments were performed in 20 counties, including just 1 treatment in Carroll County. As previously noted, few data points lead to larger errors in heat maps [30]. No data were available for 6 counties (Figure 3), including 3 (Harrison, Noble, and Monroe) that ranked among the 5 least populous counties in Ohio in 2011 [28].

**Figure 4:**
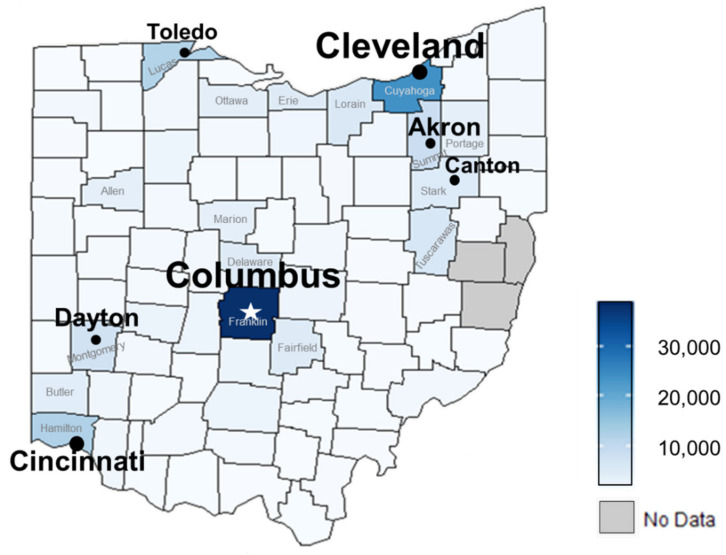
Heat map of estimated total number of bed bug treatments performed by licensed companies in each Ohio county during 2016. The darkest shade of blue indicates a maximum threshold of approximately 38,000 treatments.

In 2016, the maximum number of treatments in one county increased to over 38,000 (Figure 4); this occurred in heavily populated Franklin County in central Ohio. Two adjacent counties, Delaware and Fairfield, both had approximately 5000 treatments. In NE Ohio, the second most populous county, Cuyahoga, had almost 24,000 treatments. The bed bug problem increased in other nearby heavily populated counties, with Summit County having almost 9000 treatments and Stark County having approximately 5000 treatments. Just to the south of Stark County, Tuscarawas County had more than 6000 treatments. In NW Ohio along Lake Erie, Lucas County had almost 12,000 treatments, which was a huge increase from 2011. Also bordering Lake Erie, Sandusky, Erie, and Lorain counties ranged from approximately 4000 to 6000 treatments. In SW Ohio, Hamilton County had more than 12,000 treatments in 2016, which represents an apparent decline compared to 2011. Montgomery County had more than 7000 treatments.

Notably, in 2016, bed bug treatments occurred in 85 of Ohio’s 88 counties, with no data available for the remaining 3 counties (Figure 4). The fewest treatments, approximately 40, were in both Monroe and Williams counties, which rank 66th and 87th in population size, respectively [28]. Otherwise, in counties not previously mentioned, treatments typically ranged from approximately 100 to 3500. Note that Jefferson and Harrison counties (near the border with the northern panhandle of West Virginia) lacked data for the entire survey period (2005 to 2016).

In 2016, Ohio survey data revealed that the top 5 counties [major city] for bed bugs were: (#1) Franklin [Columbus], (#2) Cuyahoga [Cleveland], (#3) Lucas [Toledo], (#4) Hamilton [Cincinnati], and (#5) Summit [Akron]. In comparison, two nationwide companies provided 2016 bed bug data based on treatments performed by their branch offices in municipal areas. Five of Ohio’s major cities were in Terminix’s 2016 list of the top 15 most bed bug-infested cities in the US, with Cleveland-Akron ranking #3, Dayton #5, Columbus #7, Cincinnati #8, and Toledo #12 [25]. Orkin’s 2016 list of the most bed bug-infested cities nationwide ranked Columbus as #5, Cincinnati #8, and Cleveland-Akron-Canton as #13 [26]. In contrast, the 2016 survey of Ohio PMPs indicated that the number of treatments (approximately 38,000) were comparable for Franklin County (Columbus) and the three combined counties that include Cleveland-Akron-Canton. The current study is expected to be more realistic since treatment data are not company specific.

Treatment data from this study show that bed bug problems can grow and spread quickly. Furthermore, urban areas are particularly impacted by bed bug infestations. This study should alert the public as well as federal, state, and local officials that bed bugs need to be dealt with quickly rather than waiting until they become an even bigger problem.

#### 3.2.2. Treatment Sites

The most bed bug treatments were in apartments/condos and single-family homes in both 2011 and 2016 (Table 1). However, PMPs performed bed bug treatments in a wide variety of additional indoor settings such as hotels/motels, nursing homes, schools/day care centers, medical facilities, office buildings, and vehicles. The “other” category had a wide variety of sites such as camp cabins, churches, libraries, storage units, and warehouses.

**Figure 5:**
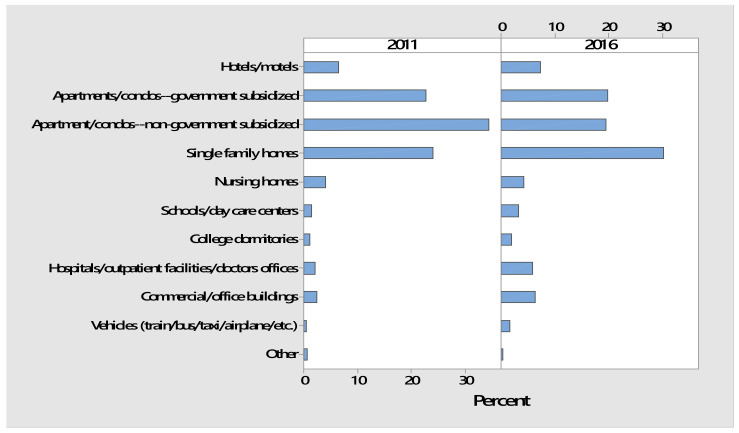
Percentages of bed bug treatment sites in 2011 and 2016.

The percentage of treatment sites significantly differed (*p <* 5.2 × 10^−13^) between 2011 and 2016 (Chi-Square Test of Independence). The bar charts in Figure 5 show that the most evident change from 2011 to 2016 was a large increase in the percentage of single-family homes and a decrease in non-government subsidized apartment/condos.

#### 3.2.3. Types of Treatments Used by PMPs

The diverse types of treatments that PMPs typically used to control bed bugs are listed in Table 2. Insecticides/chemicals were most frequently used by PMPs in both 2011 and 2016. Nonetheless, all respondents said that they used a combination of the listed treatments, which represents an IPM approach for bed bugs. Hence, the current study further documents PMPs adoption of IPM for urban pests, which was found to be a general trend within the US pest management industry in the late 1990s [34]. Insecticides have long been a mainstay for treating bed bugs [5,14,15,16,35,36], although these insects quickly develop resistance to insecticides [13,14,15,16,37]. Bed bug populations in the US have shown resistance or reduced susceptibility to pyrethroids [37,38,39,40,41] and newer insecticide classes such as neonicotinoids and pyrroles [42].

The least used treatment approaches were placing items into freezers and structural fumigation (Table 2). Heat kills bed bugs much quicker and tends to be more reliable than the fluctuating cold temperatures of household freezers [43]. Structural fumigation is the most expensive treatment type; it is imperative to have trained personnel and special equipment due in part to the extreme inhalation hazard posed by fumigants. Sulfuryl fluoride, a restricted-use pesticide, is currently used for structural fumigations in the US [44]. Fumigation requires a distinct license in Ohio (Category 10c) and other states.

**Figure 6:**
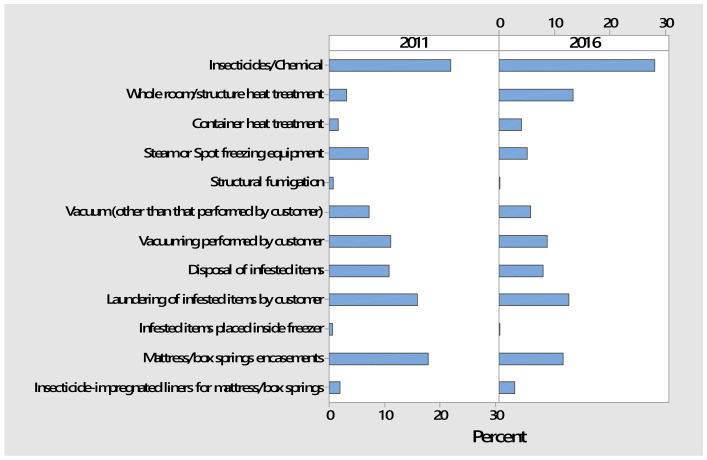
Percentages of treatment types used by PMP respondents in 2011 and 2016.

The proportion of treatment types significantly differed between 2011 and 2016 (*p <* 2.2 × 10^−16^, Chi-Square Test of Independence). The most evident change was that whole room/structure heat treatment greatly increased and mattress/box springs encasements apparently decreased (Figure 6). In 2011, commercial equipment for heat treatments was still relatively new to the marketplace [14,45]. However, heat treatments subsequently became a more widely used practice by PMPs, with numerous companies specializing in this approach. Structural heat treatments require special equipment and trained personnel to ensure that lethal temperatures are achieved throughout a structure while being safely applied [46]. Heat treatment offers advantages such as reduced use of insecticide indoors, faster efficacy, ability to treat diverse articles, and reduced effort processing electronics and infested laundry [47]. This may help explain the decrease in laundering performed by customers in the 2016 survey. Since heat provides no residual effects, it often is supplemented with targeted insecticide/chemical treatment, which was the most frequently used treatment type in both surveys. There is no clear reason for the decrease in mattress/box springs encasements in 2016.

#### 3.2.4. Number of PMP Visits Per Infestation

**Figure 7:**
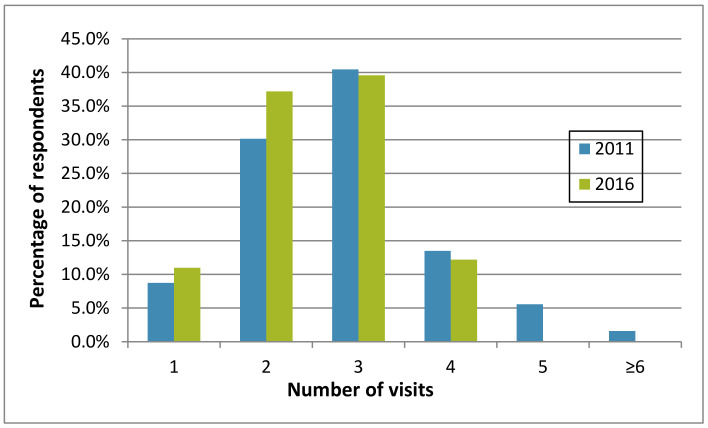
Number of treatment visits (1–≥6) to eliminate a bed bug infestation according to survey respondents in 2011 and 2016.

In both surveys, the question was posed: “On average, how many total visits per infestation are needed to achieve bed bug control?” Most licensed companies reported that two to three visits were needed to eliminate an infestation in both 2011 and 2016 (Figure 7). However, the range decreased over time, with no respondents in 2016 saying that five or more visits were needed to achieve bed bug control. Presumably, this resulted from a larger number of companies having more experience treating for bed bugs and their use of more effective products. Few companies reported that only a single visit was needed for bed bug control (8.7% in 2011 and 11.0% in 2016; Figure 7). In a 2005 survey of PMPs nationwide, only 6.1% claimed successful bed bug control after a single treatment [3].

#### 3.2.5. Detection Methods Used by PMPs

Confirmation of bed bugs is a critical step in deciding whether to initiate treatment. Respondents provided information on their use of common bed bug detection methods (visual inspection, passive monitors, active monitors, and canines [2016 survey only]). Bed bug detection using each of these methods is addressed in the USA’s National Pest Management Association (NPMA) Best Management Practices for Bed Bugs (BMPs) [48]. These guidelines were developed by industry professionals, regulators, academics, and entomologists.

The vast majority of Ohio PMPs indicated that they always or often used visual inspection to help diagnose bed bugs (98.5% in 2011 and 96.5% in 2016). Other surveys have reported similar findings [5,6,7,8,10]. Visual inspection depends on finding bed bugs of any stage (egg, nymph, adult) and/or bed bug signs such as fecal deposits and shed skins [20,48,49].

Visual detection based solely on bed bug bites is unreliable. Some people have no skin reactions, particularly those who are older (>65 y) [49]. This finding is further supported by interviews of elderly residents living in infested apartments [19,50]. Some others experience red welts or redness/skin discoloration, with or without itchiness [20,49,51,52]. Bed bug bites sometimes appear to have a linear or grouped pattern, with divergent opinions on whether the bite pattern is diagnostic [53] or unreliable [52]. The NPMA BMPs [48] offer the following guidance:

*“9.7. The presence of skin reactions or assurances by residents that bed bugs are present should be considered carefully.*

*9.7.1. It is not possible to tell from an apparent bite if it was caused by a bed bug because skin reactions vary, and skin reactions from other insects may have similar appearance to those of bed bugs.*

*9.7.2. Skin infections and conditions can also look like insect bites.”*

Passive monitoring devices tend to be inexpensive options for bed bug detection, but their efficacy can vary widely. Glue boards and sticky traps decreased as always- or often-used-devices by Ohio PMPs from 38.5% in 2011 to 18.9% in 2016. In 2005, 67.9% of PMPs nationwide reported using sticky traps [3]. Research has shown that sticky traps are ineffective in capturing bed bugs [54].

Pitfall traps also are passive monitors, and they typically rely on their smooth inner surface to prevent the escape of trapped bugs. Pitfall traps were not widely used by Ohio PMPs. Respondents indicated that they never or rarely used them (65.1% in 2011 and 63.5% in 2016). A minority of respondents always or often used pitfall traps, with some increase from 2011 (12.7%) to 2016 (19.5%). An optimal position for pitfall traps is under furniture legs [55]. Several types of pitfall traps were found to be effective in detecting and monitoring *C. lectularius* [55,56,57]. In a recent study, two commercially available pitfall traps (ClimbUp Insect Interceptors and BlackOut BedBug Detectors) demonstrated high detection rates with 1, 2, or 4 traps per apartment; however, it was important to situate one trap near the bed when placing only a few monitors [54].

Active monitoring devices (traps using heat and/or chemical attractants such as carbon dioxide or pheromones) were relatively new to the marketplace in 2011, and at that time, few respondents indicated that they always or often used these devices (9.7%). Their use more than doubled by 2016, with 24.4% of respondents always or often using active monitoring devices. Research in occupied high-rise apartments showed that prototype Verifi Bed Bug Detectors (3 per room) provided high bed bug detection rates when situated near frequently used furniture [58]. In contrast, Verifi Bed Bug Detectors were found to be ineffective in determining the bed bug burden in a Cleveland, Ohio emergency room [59]. However, their study design appears flawed because too few traps (6 total) were deployed in an apparently large area with more than 30 rooms (no dimensions reported), the traps were situated outside of patient rooms when bed bugs preferentially occupy sites near human hosts, and lures were replaced at 4 months rather than the specified ≤ 90 d interval. (Verifi Bed Bug Detectors and components are no longer commercially available due to the company’s marketing decision.)

The 2016 survey respondents indicated that canine detection was not widely used in Ohio. Only 10.1% of respondents always or often used canines; 71.1% said that they never used them. In 2010, 15% of companies surveyed nationwide used trained dogs for detecting bed bugs [5]. Although canines have a particularly good detection record in controlled setting [49,60], their record in real-world settings is quite variable [14,61,62].

### 3.3. Residents’ Responses to Bed Bugs

Ohio PMPs provided information on their customers’ misidentification of bed bugs, use of DIY pest control products, and furniture disposal. The surveys sought PMPs’ recall of their customers’ actions in the recent past (2011 and 2016), not their general impressions of the public.

#### 3.3.1. Bed Bug Misidentification by Residents

In both surveys, PMPs provided information on the most common insects/things that their customers misdiagnosed as bed bugs. Open-ended responses in the 2011 survey provided for a huge variety of things such as bat bugs, carpet beetles, ticks, biting insects, flea bites, feeling of being bit, dirt specks, debris, and lint (140 respondents). Responses ranged from very general to extremely specific (e.g., beetles versus drug store beetles; roaches versus German cockroach nymphs; mites versus chiggers).

Fixed responses in the 2016 survey are detailed in Table 3 for respondents who selected one or more items from seven choices. Most respondents selected carpet beetles (adults, larvae, or shed skins) and non-insects (skin scrapings, lint, debris, etc.). Carpet beetles were ranked as the #1 most misdiagnosed. Cockroaches and bat bugs were next most frequently misdiagnosed as bed bugs. Less commonly misdiagnosed were stored product beetles, stink bugs, and “other.” The latter category was selected by 28 respondents who listed a total of 18 different things. Fleas were listed by the most respondents (n = 10). This was followed by ticks (n = 3), multicolored Asian lady beetles (n = 2), scabies (n = 2), and delusory parasitosis/phobia (n = 2). Singular responses included such things as ants, aphids, crab lice, millipedes, moths, and no-see-ums.

PMP respondents estimated that approximately 15% of customers asked for bed bug treatment when told that they had a different pest. Irrational fear of bed bugs often plays a role in such requests. People also may incorrectly attribute an undiagnosed skin reaction to bed bugs. Furthermore, the public does not necessarily understand that insects have diverse behaviors and habitats, and treatment needs to be tailored to each pest. More public education is needed to address such issues.

#### 3.3.2. Do-It-Yourself (DIY) Bed Bug Control by Residents

**Figure 8:**
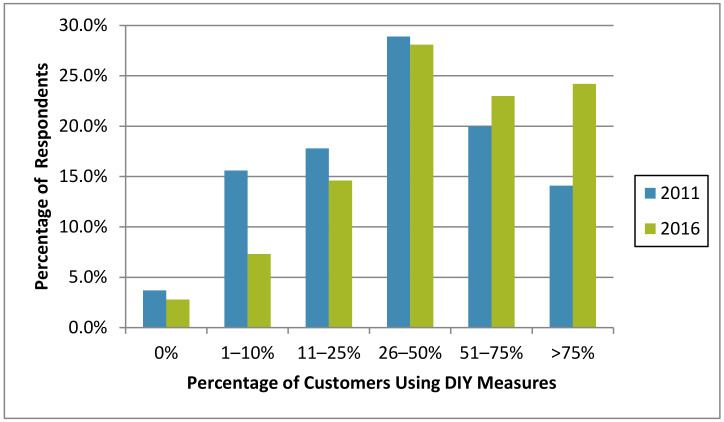
Estimated percentage of customers using DIY measures before calling for a licensed professional in 2011 and 2016.

Many companies estimated that more than half of their customers initially used DIY measures against bed bugs before calling for professional services, with this perception increasing from 2011 to 2016 (34.1% and 47.2% of respondents, respectively; Figure 8). This is similar to the perspective of PMPs nationwide—in 2010, 51% of U.S. PMPs estimated that 50% or more of their customers attempted DIY bed bug control before calling a professional [5]. A 2013 survey in Philadelphia, Pennsylvania, USA, noted that 69.2% of households self-reported attempting to eliminate bed bugs without professional assistance [63]. A myriad of factors may be related to residents’ reliance on DIY measures. Some do not report a bed bug problem because of embarrassment, fear of eviction, the expense of paying for treatment, or failure to recognize the pest [51].

In the 2016 survey, when asked about customer success using DIY measures, the majority of Ohio PMPs thought that none of their customers were able to solve their bed bug infestation (62.1%). Some PMPs thought that approximately 1 to 10% of their customers were successful (28.0%), particularly when they were dealing with only a few bed bugs, but success was never achieved in a heavily infested residence. A few respondents estimated high success (70 to 90%) but typically specified that it was critical for customers to obtain effective products (not over-the-counter [OTC] products) as well as professional advice on how and where to apply products; early detection and minimal clutter also were essential.

PMPs perceptions of treatment success sharply contrast with some peoples’ perceptions. For example, in a Philadelphia survey, 44.4% of households perceived that they eliminated bed bugs after solely using self-treatment measures [63]. Even with professional treatment, 76% of residents thought that their apartment was no longer infested even though bed bugs continued to be detected [50].

In the Ohio surveys, approximately half of respondents felt that customers stopped DIY measures once professional services began, but the other half felt that some customers continued with DIY contrary to PMP instructions. Continued DIY efforts can counteract the non-chemical and chemical treatment measures implemented by professionals and lengthen the time for bed bug elimination.

In both surveys, Ohio companies were asked to estimate their customers’ spending on DIY bed bug products/treatments. In 2011, customers were estimated to spend approximately $171 ± $158 (mean, SD; US dollars), with a mode of $100 and a maximum of $1200. In 2016, estimated DIY cost for customers was approximately $230 ± $430 (mean, SD) with a mode of $100 and a maximum of $5000. These estimates are comparable to findings from the 2013 Philadelphia survey—households that solely self-treated for bed bugs reported spending from $10 to $800 (mean $229.10) [63].

#### 3.3.3. Product Misuse by Residents

Ohio residents misused many products in their attempts to combat bed bugs. Misused products included a wide variety of pesticides as well as chemicals commonly found in and around the home or yard (Table 4). Likewise, PMPs nationwide reported seeing many of these same ineffective and potentially dangerous measures [5]. In that 2010 survey, U.S. PMPs felt that their customers with bed bugs were “not very” (31%) or “not at all” (62%) concerned about applying insecticides. The U.S. Centers for Disease Control and Prevention (CDC) documented 111 cases of poisoning associated with pesticide misuse by individuals treating for bed bugs in 7 states during the period 2003 to 2010 [64].

**Figure 9:**
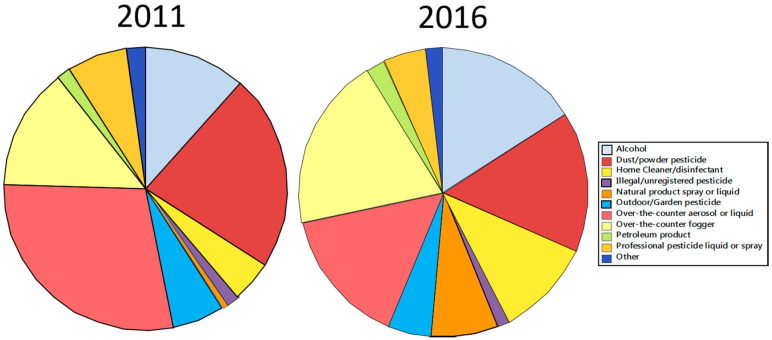
Proportion of products misused by residents in 2011 and 2016.

The proportion of products misused by residents significantly differed between 2011 and 2016 (*p* < 8.2 × 10^−6^). Pie charts for both years show that the most evident change from 2011 to 2016 was increased misuse of bug bombs and alcohol, with decreased misuse of OTC aerosol or liquid pesticides and dust/powder pesticides (Figure 9). Not only are OTC foggers ineffective against bed bugs [65], but their chemical residues are deposited on surfaces that humans frequently contact [66]. Another huge downside is that bug bombs cause bed bugs to spread, even to adjacent apartments. Alcohol is ineffective against bed bugs unless they drown in the liquid. Spraying alcohol on one’s shoes, luggage, etc., is ineffective because alcohol quickly evaporates and does not kill the bugs on contact. Furthermore, alcohol is highly flammable and can be dangerous if applied in large quantities or aerosolized.

PMPs are in a unique position to know about product use and misuse by residents. For example, when formulating their work plan, PMPs typically query residents as to prior pesticide use. For their own protection, PMPs also need to ascertain what products previously have been used, particularly when dusts, powders, or residues are visible on indoor surfaces. Furthermore, as PMPs go about treating for bed bugs, they see product containers in living areas, cabinets, trash cans, etc.

#### 3.3.4. Furniture Disposal by Residents

**Figure 10:**
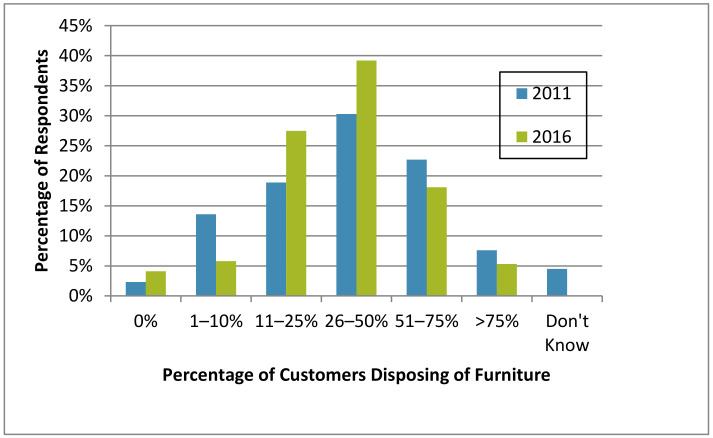
Estimated percentage of customers discarding infested furniture in in 2011 and 2016.

Most survey respondents in 2011 and 2016 thought that their customers discarded infested furniture to some extent (Figure 10). This perception slightly increased from 2011 to 2016 (93.1% and 95.9% of respondents, respectively). In a high-rise apartment building in Indianapolis, Indiana, USA, 35% of surveyed residents in 2010 reported that they discarded furniture [19].

Not only is furniture expensive, but replacement furniture can be quickly infested by other bed bugs remaining in a residence. More importantly, throwing away infested furniture increases the risk of spreading bed bugs on site and to others. If infested furniture is unwanted, unsalvageable, or otherwise requires disposal, it first should be treated to kill as many bed bugs as possible, then it should be damaged or defaced to make it unusable so that others are not tempted to bring it into their residences. It is a good idea to clearly label the item as having bed bugs. To prevent any remaining bed bugs from falling off while the furniture is moved outdoors, it needs to be securely wrapped in plastic sheeting (large items) or securely enclosed in a bag (small items). Infested furniture should be kept wrapped while awaiting pickup and disposal. Ideally, it should be taken to the dump by the customer. In both surveys, PMPs thought that most customers never or rarely followed any of these steps for proper furniture disposal.

## 4. Conclusions

The magnitude and spread of bed bug infestations in Ohio were demonstrated by treatment records of licensed PMPs. Data from 2005 indicated that Ohio’s bed bug problem likely started in the SW corner of the state in Hamilton County (includes city of Cincinnati), which incurred an estimated total of 4500 bed bug treatments. Numerous other less populous counties in SW Ohio also experienced bed bug treatments during 2005. The magnitude of the bed bug problem in Ohio greatly increased in subsequent years, with a maximum of approximately 17,000 total treatments during the first half of 2011 and approximately 38,000 total treatments during 2016. Bed bug problems expanded statewide from 7 counties, with 100+ total treatments in 2005, to 45 counties in 2011, to nearly all 88 counties in 2016. Ohio counties with large metropolitan areas were the most heavily infested with bed bugs. The bed bug problem was exacerbated by residents’ behaviors that included misidentifying bed bugs, attempting DIY pest control, misusing OTC products, and discarding infested furniture. More public education is needed to stop such practices. This study shows that bed bug problems grow and spread quickly; federal, state, and local officials and the public should immediately deal with bed bugs.

## Figures and Tables

**Table 1:** Sites treated by licensed PMPs in 2011 and 2016.

Treatment Site	Frequency of Responses ^1^	
	2011	2016
Hotels/motels	2117	4743
Apartments/condos—government subsidized	7457	12,748
Apartment/condos—non-government subsidized	11,290	12,500
Single-family homes	7852	19,411
Nursing homes	1319	2689
Schools/day care centers	498	2061
College dormitories	355	1289
Hospitals/outpatient facilities/doctors’ offices	691	3794
Commercial/office buildings	802	4084
Vehicles (train/bus/taxi/airplane/etc.)	160	1061
Other	195	178

^1^ Respondents had the option of selecting one or more treatment sites.

**Table 2:** Types of treatments used by Ohio’s licensed PMPs for bed bug management in 2011 and 2016.

Type of Treatment ^1,2^	Frequency of Responses	
	2011	2016
Insecticides/Chemical ^3^	125	297
Whole room/structure heat treatment	18	142
Container heat treatment	9	43
Steam or spot freezing equipment	40	54
Structural fumigation	4	2
Vacuum (other than that performed by customer)	41	60
Vacuuming performed by customer	63	92
Disposal of infested items	62	85
Laundering of infested items by customer	91	134
Infested items placed inside freezer	3	1
Mattress/box springs encasements	102	122
Insecticide-impregnated liners for mattress/box springs	11	31

^1^ Respondents had the option of selecting one or more treatment types. ^2^ In order to keep the same number of categories, the ‘Other’ option in the 2016 survey (selected by 1.0% of respondents) is not listed. Physical removal using traps was most frequently mentioned. ^3^ In order to make the two years comparable, two categories, ‘Insecticide/Chemical sprays’ and ‘Insecticide/Chemical dusts,’ in the 2016 survey were combined into one category, ’Insecticide/Chemical.’.

**Table 3:** Insects or things that customers misdiagnosed as bed bugs according to 2016 survey respondents.

Insects/Things Misdiagnosed as Bed Bugs	Frequency of Responses ^1,2^	Frequency of Rank #1 ^3^
Carpet beetles (adults, larvae, or shed skins)	79	37
Cockroaches	47	10
Stink bugs	26	2
Stored product beetles	33	0
Bat bugs	41	8
Non-insects (skin scrapings, lint, debris, etc.)	80	29
Other	28	14

^1^ Respondents had the option of selecting one or more misdiagnosed things from list of 7 choices. ^2^ Total of 128 respondents. ^3^ Total of 102 respondents provided ranking.

**Table 4:** Products misused by residents in 2011 and 2016 according to Ohio PMPs.

Products Misused by Residents	Frequency of Responses ^1^	
	2011	2016
Alcohol (isopropyl, rubbing, etc.)	22	113
Dust/powder pesticide	42	112
Home cleaner/disinfectant	9	78
Illegal/unregistered pesticide	3	10
Natural product spray or liquid	1	53
Outdoor/garden pesticide	11	34
Over-the-counter aerosol or liquid	54	111
Over-the-counter fogger (bug bombs)	26	139
Petroleum product	3	15
Professional pesticide liquid or spray	13	34
Other	4	14
Total	188	713

^1^ Respondents had the option of selecting one or more misused products.

## Data Availability

The data presented in this study are available on request from the corresponding author. The data are not publicly available due to anticipated publication of one or two related articles in technical magazines for the pest management industry, and these data are part of a much larger dataset not yet published in its entirety.

## Supplementary Materials

The following are available online at https://www.mdpi.com/2075-4450/12/2/133/s1, Exhibit S1: 2011 Ohio Bed Bug Survey Questionnaire; Exhibit S2: 2016 Ohio Bed Bug Survey Questionnaire; and Exhibit S3: List of Participating Companies.
