# Supplementary material for: Magnitude and Spread of Bed Bugs (Cimex lectularius) throughout Ohio (USA) Revealed by Surveys of Pest Management Industry
Source: Insects. 2021 Feb 4;12(2):133. doi: 10.3390/insects12020133 (PMC7913827; doi:10.3390/insects12020133)
Supplement: Supplementary file 1 [file insects-12-00133-s001.zip › Exhibit S1--2011 OH Bed Bug Survey Questionnaire.pdf]

Cover Page

Ohio Statewide Bed Bug Survey

1.

1. **In which Ohio county is your business located? If your company has more than one branch, please answer only for the branch at which you are located. If you are located out-of-state, please circle 89-Out-of-State. (*please circle ONE ONLY)***

1-Adams	16-Coshocton	31-Hamilton	46-Logan	61-Noble	76-Stark
2-Allen	17-Crawford	32-Hancock	47-Lorain	62-Ottawa	77-Summit
3-Ashland	18-Cuyahoga	33-Hardin	48-Lucas	63-Paulding	78-Trumbull
4-Ashtabula	19-Darke	34-Harrison	49-Madison	64-Perry	79-Tuscarawas
5-Athens	20-Defiance	35-Henry	50-Mahoning	65-Pickaway	80-Union
6-Auglaize	21-Delaware	36-Highland	51-Marion	66-Pike	81-Van Wert
7-Belmont	22-Erie	37-Hocking	52-Medina	67-Portage	82-Vinton
8-Brown	23-Fairfield	38-Holmes	53-Meigs	68-Preble	83-Warren
9-Butler	24-Fayette	39-Huron	54-Mercer	69-Putnam	84-Washington
10-Carroll	25-Franklin	40-Jackson	55-Miami	70-Richland	85-Wayne
11-Champaign	26-Fulton	41-Jefferson	56-Monroe	71-Ross	86-Williams
12-Clark	27-Gallia	42-Knox	57-Montgomery	72-Sandusky	87-Wood
13-Clermont	28-Geauga	43-Lake	58-Morgan	73-Scioto	88-Wyandot
14-Clinton	29-Greene	44-Lawrence	59-Morrow	74-Seneca	89-Out-of-State
15-Columbiana	30-Guernsey	45-Licking	60-Muskingum	75-Shelby	

2. **What year was your company (branch office) established/licensed in Ohio?** (*please enter 4-digit year)*

3. **How many employees does your company (branch office) currently have?** *(please enter number)*

4. **Do you provide treatment services for bed bugs? *(please circle)***

Yes **No** *(If No, please skip to Question 30)*

5. In which Ohio counties do you perform bed bug work? *(please circle each that is applicable)*
Please indicate the approximate percentage of your bed bug work that is done in each county that you selected; you should insert the percentage in the box located <u>after</u> each selected county. (*Please note that all the percentages in the table must sum to 100%.*)

1-Adams		19-Darke		37-Hocking		55-Miami		73-Scioto	
2-Allen		20-Defiance		38-Holmes		56-Monroe		74-Seneca	
3-Ashland		21-Delaware		39-Huron		57-Montgomery		75-Shelby	
4-Ashtabula		22-Erie		40-Jackson		58-Morgan		76-Stark	
5-Athens		23-Fairfield		41-Jefferson		59-Morrow		77-Summit	
6-Auglaize		24-Fayette		42-Knox		60-Muskingum		78-Trumbull	
7-Belmont		25-Franklin		43-Lake		61-Noble		79-Tuscarawas	
8-Brown		26-Fulton		44-Lawrence		62-Ottawa		80-Union	
9-Butler		27-Gallia		45-Licking		63-Paulding		81-Van Wert	
10-Carroll		28-Geauga		46-Logan		64-Perry		82-Vinton	
11-Champaign		29-Greene		47-Lorain		65-Pickaway		83-Warren	
12-Clark		30-Guernsey		48-Lucas		66-Pike		84-Washington	
13-Clermont		31-Hamilton		49-Madison		67-Portage		85-Wayne	
14-Clinton		32-Hancock		50-Mahoning		68-Preble		86-Williams	
15-Columbiana		33-Hardin		51-Marion		69-Putnam		87-Wood	
16-Coshocton		34-Harrison		52-Medina		70-Richland		88-Wyandot	
17-Crawford		35-Henry		53-Meigs		71-Ross			
18-Cuyahoga		36-Highland		54-Mercer		72-Sandusky			

6. **In which of the following sites does your company (branch) treat for bed bugs, and approximately what percentage of your overall bed bug work is done in each? (*Please note that percentages must sum to 100%.*)**

Site	%	Site	%
Hotels/motels		College dormitories	
Apartments/condos--government subsidized		Hospitals/outpatient facilities/doctor's offices	
Apartment/condos--non-government subsidized		Commercial/office buildings	
Single family homes		Vehicles (train/bus/taxi/airplane/etc.)	
Nursing homes		Other	
Schools/day care centers			

7. **Do you have call tracking?** ***(please circle one)*** **Yes** **No**

8. **How many total calls did you receive regarding treatment for bed bugs during each of the following years (2005-2011)? Note, if you do not have call tracking, please estimate the number of calls.** ***(Please check one category for each year.)***

# Calls / Year	0	1-10	11-50	51-100	101-500	501-1000	1001-2000	2001-3000	3001-4000	4001-5000	>5000	Don't Know
2005	☐	☐	☐	☐	☐	☐	☐	☐	☐	☐	☐	☐
2006	☐	☐	☐	☐	☐	☐	☐	☐	☐	☐	☐	☐
2007	☐	☐	☐	☐	☐	☐	☐	☐	☐	☐	☐	☐
2008	☐	☐	☐	☐	☐	☐	☐	☐	☐	☐	☐	☐
2009	☐	☐	☐	☐	☐	☐	☐	☐	☐	☐	☐	☐
2010	☐	☐	☐	☐	☐	☐	☐	☐	☐	☐	☐	☐
2011 (through June 30)	☐	☐	☐	☐	☐	☐	☐	☐	☐	☐	☐	☐

9. **Approximately what percentage of your customers that request bed bug work actually have a pest other than bed bugs (misdiagnosed by customers)?** ***(please round to nearest whole percent)*** ☐

10. **What are the most common insects or things that your customers have misdiagnosed as bed bugs?**

____

____

____

11. **In your estimation, what percentage of your customers have attempted do-it-yourself treatment(s) for bed bugs?** ***(Please circle one from each row)***

**Before hiring you**	0%	1-10%	11-25%	26-50%	51-75%	>75%
**While you are still conducting the customer's bed bug job**	0%	1-10%	11-25%	26-50%	51-75%	>75%

12. **If you answered a percentage greater than 0% above, how much do you estimate that your customers spend, on average per year, for do-it-yourself bed bug products/treatments?** ***(please round to nearest dollar)*** ***$*** ☐

13. **In your estimation, what percentage of your customers have misused products in an attempt to kill bed bugs? *(please circle one)***

0% 1-10% 11-25% 26-50% 51-75% >75%

14. **If you are aware of customers misusing products for bed bugs, what are the three most common products being misused?**

Product 1 ______

Product 2 ______

Product 3 ______

15. **What percentage of your customers have disposed of their furniture in their efforts to control bed bugs? *(please circle one)***

0% 1-10% 11-25% 26-50% 51-75% >75%

16. **If you answered a percentage greater than 0% above, to what extent are your customers using the following procedures to dispose of bed bug-infested furniture? *(please check one per row)***

	Never	Rarely	Sometimes	Often	Always
Items are wrapped prior to disposal	☐	☐	☐	☐	☐
Items are treated prior to disposal	☐	☐	☐	☐	☐
Prior to disposal, items are ripped, damaged, or otherwise defaced to prevent re-use	☐	☐	☐	☐	☐
Items are placed for normal trash pick-up	☐	☐	☐	☐	☐
Items are taken to the dump by the customer or their designee	☐	☐	☐	☐	☐
Items are given away or sold to someone else	☐	☐	☐	☐	☐

17. **To what extent is your company using the following methods/devices to detect bed bugs? (*please check one per row*)**

	Never	Rarely	Sometimes	Often	Always
Visual inspection	☐	☐	☐	☐	☐
Active monitoring devices (traps using heat and/or carbon dioxide [CO_2])	☐	☐	☐	☐	☐
Glueboards or sticky traps	☐	☐	☐	☐	☐
Pitfall traps (typically placed underneath each bed leg)	☐	☐	☐	☐	☐

18. **How many treatments for bed bugs (individual residence, individual unit or apartment treated within an apartment building, company office treated within a larger building, hotel room, etc.) did your company (branch office) conduct during each of the following years (2005-2011)? Note, if you do not have exact numbers, please estimate the number of treatments.** ***(Please check only one box for each year)***

# Calls / Year	0	1-10	11-50	51-100	101-500	501-1000	1001-2000	2001-3000	>3000	Don't Know	Did not provide trtmts
**2005**	☐	☐	☐	☐	☐	☐	☐	☐	☐	☐	☐
**2006**	☐	☐	☐	☐	☐	☐	☐	☐	☐	☐	☐
**2007**	☐	☐	☐	☐	☐	☐	☐	☐	☐	☐	☐
**2008**	☐	☐	☐	☐	☐	☐	☐	☐	☐	☐	☐
**2009**	☐	☐	☐	☐	☐	☐	☐	☐	☐	☐	☐
**2010**	☐	☐	☐	☐	☐	☐	☐	☐	☐	☐	☐
**2011 (through June 30)**	☐	☐	☐	☐	☐	☐	☐	☐	☐	☐	☐

19. **On average, how many total visits per infestation are needed to achieve bed bug control?** ***(Please circle one)***

0 1 2 3 4 5 >5

20. **What type of treatments/products do you typically use to control bed bugs?** ***(Check all that apply)***

☐ 1-Insecticides/Chemical
☐ 2-Whole room/structure heat treatment
☐ 3-Container heat treatment
☐ 4-Steam
☐ 5-Spot freezing equipment
☐ 6-Structural fumigation
☐ 7-Vacuum (other than that performed by customer)
☐ 8-Vacuuming performed by customer
☐ 9-Disposal of infested items
☐ 10-Mattress/box springs encasements
☐ 11-Infested items placed inside freezer
☐ 12-Laundering of infested items by customer (using washer +/or clothes drier)
☐ 13-Insecticide-impregnated liners for mattress/box springs

21. **If insecticides/chemical checked above, please list all chemicals used in 2011? If possible, please include the chemical name with the product name.**

____

____

____

22. **How would you rate your level of satisfaction with the <u>currently available chemical treatment options</u> for bed bugs *(please circle one)*?**

1 -Very Dissatisfied	2 - Dissatisfied	3 - Neither Dissatisfied nor Satisfied	4 - Satisfied	5 - Very Satisfied

Please provide comments as to why you are satisfied or dissatisfied with the currently available chemical treatment options?

23. **How would you rate your level of satisfaction with the <u>currently available non-chemical treatment options</u> for bed bugs *(please circle one)*?**

1 -Very Dissatisfied	2 - Dissatisfied	3 - Neither Dissatisfied nor Satisfied	4 - Satisfied	5 - Very Satisfied

Please provide comments as to why you are satisfied or dissatisfied with the currently available non-chemical treatment options?

24. **Currently, what percentage of your total treatments fall into the following categories? *(Please note that percentages must sum to 100%.)***

Number of Bed Bugs	Percent of Total Treatments
< 10 bed bugs per infestation (light infestation)	%
11-100 bed bugs per infestation (medium infestation)	%
101-1000 bed bugs per infestation (heavy infestation)	%
>1000 bed bugs per infestation (very heavy infestation)	%

25. **Is it your policy to inspect surrounding units (when possible) within a multi-family dwelling?** ***(please circle one*)**

Yes No Don't know Not applicable

26. **Do you feel that your customers are recognizing bed bug infestations earlier than in the past?** ***(please circle one*)**

Yes No Don't know

27. **Do you provide retail items (for bed bugs)?** ***(please circle one)***

Yes No Don't know

28. **If you answered Yes to question 27 above: What retail products do you offer?**

____

____

____

29. **Please provide any additional comments or suggestions regarding bed bugs that you would like to share, or additional clarification of particular answers in this survey.**

____

____

____

The following three items are optional, but known survey participants will be acknowledged, primarily by company name, in presentations and publications outlining the survey results. If you wish to be publicly acknowledged for your help with the survey, please provide the following information. And, THANK YOU for your help with this important endeavor.

30. Your name (optional) ____

31. Your company (optional) ____

32. Your email (optional) ____

Please use the enclosed envelope to return this questionnaire at no cost to you. You may also mail the questionnaire to:

Statistical Consulting Service
The Ohio State University
328 Cockins Hall
1958 Neil Avenue
Columbus OH 43210
