# Supplementary material for: Magnitude and Spread of Bed Bugs (Cimex lectularius) throughout Ohio (USA) Revealed by Surveys of Pest Management Industry
Source: Insects. 2021 Feb 4;12(2):133. doi: 10.3390/insects12020133 (PMC7913827; doi:10.3390/insects12020133)
Supplement: Supplementary file 1 [file insects-12-00133-s001.zip › Exhibit S2--2016 OH Bed Bug Survey Questionnaire.pdf]

2016 Ohio Statewide Bed Bug Survey

Dear Colleague:

Like many states, Ohio has been considerably impacted by bed bugs, but the extent of the bed bug issue has not been adequately documented or tracked throughout the state. This Ohio Statewide Bed Bug Survey is a research project for gathering such data.

This survey is being disseminated only to pest management companies/individuals who are licensed (category 10A) to treat for bed bugs in the state of Ohio. (Because of the type of data to be collected, we ask that only pest management professionals complete the survey.) Your participation is voluntary. Furthermore, participants can skip any questions they wish not to answer.

Even if you don't do bed bug work, we need to know this! If you don't do bed bug work, then only the first four brief survey items pertain to you.

To ensure that your data remain anonymous, any identifiers such as your name, address, email will be stripped from the survey tallies. De-identified data will be maintained for a period of at least 5 years after study completion per the Ohio State Research Data policy.

At the conclusion of the survey, we would like to publicly acknowledge the participants. There are two options if you wish to be acknowledged: you can provide your name, company, and email (survey items 30-32, which are on a separate page), or you can skip items 30-32 and contact me at jones.1800@osu.edu to let me know that you completed the survey.

For questions about your rights as a participant in this study or to discuss other study-related concerns or complaints with someone who is not part of the research team, you may contact Ms. Sandra Meadows in the Office of Responsible Research Practices at 1-800-678-6251 or hsconcerns@osu.edu.

Plans are to share the survey results at future meetings and in a journal or trade magazine. Pertinent data summaries will be shared with EPA and HUD in an effort to increase federal recognition of the bed bug problem.

We ask for your immediate assistance in completing the following survey. ***Your feedback is requested no later than Tuesday, March 21, 2017.***

THANK YOU for your help with this important endeavor. Your support is very much appreciated.

Dr. Susan C. Jones

Urban Entomologist &
State Extension Specialist on Household and Structural Insects

Note: Funding for the Ohio Statewide Bed Bug Survey has been provided by a grant to OSU Extension Entomology from the USDA National Institute of Food and Agriculture, Extension IPM Program.

Additional Instructions: If your company has more than one branch office, please answer the survey questions only for the branch at which you are located or with the help of regional/district/other branch managers; please fill out a separate survey for each location.

Ohio Statewide Bed Bug Survey

1. **In which Ohio county is your business located? If your company has more than one branch, please answer only for the branch at which you are located. If you are located out-of-state, please circle 89-Out-of-State. (*please circle ONE ONLY)***

1-Adams	16-Coshocton	31-Hamilton	46-Logan	61-Noble	76-Stark
2-Allen	17-Crawford	32-Hancock	47-Lorain	62-Ottawa	77-Summit
3-Ashland	18-Cuyahoga	33-Hardin	48-Lucas	63-Paulding	78-Trumbull
4-Ashtabula	19-Darke	34-Harrison	49-Madison	64-Perry	79-Tuscarawas
5-Athens	20-Defiance	35-Henry	50-Mahoning	65-Pickaway	80-Union
6-Auglaize	21-Delaware	36-Highland	51-Marion	66-Pike	81-Van Wert
7-Belmont	22-Erie	37-Hocking	52-Medina	67-Portage	82-Vinton
8-Brown	23-Fairfield	38-Holmes	53-Meigs	68-Preble	83-Warren
9-Butler	24-Fayette	39-Huron	54-Mercer	69-Putnam	84-Washington
10-Carroll	25-Franklin	40-Jackson	55-Miami	70-Richland	85-Wayne
11-Champaign	26-Fulton	41-Jefferson	56-Monroe	71-Ross	86-Williams
12-Clark	27-Gallia	42-Knox	57-Montgomery	72-Sandusky	87-Wood
13-Clermont	28-Geauga	43-Lake	58-Morgan	73-Scioto	88-Wyandot
14-Clinton	29-Greene	44-Lawrence	59-Morrow	74-Seneca	89-Out-of-State
15-Columbiana	30-Guernsey	45-Licking	60-Muskingum	75-Shelby	

2. **What year was your company (branch office) established/licensed in Ohio?** (*please enter 4-digit year*) [ ]

3. **How many employees does your company (branch office) currently have?** *(please enter number)* [ ]

4. **Do you provide treatment services for bed bugs in Ohio? *(please circle)***

Yes **No** *(If No, thank you! You have finished the 2017 survey.)*

5. **In which Ohio counties do you perform bed bug work?** ***(Please circle each that is applicable.)*** **Please indicate the approximate percentage of your bed bug work that is done in each county that you selected; you should insert the percentage in the box located <u>after</u> each selected county. (*Please note that all the percentages in the table must sum to 100%.)***

1-Adams		19-Darke		37-Hocking		55-Miami		73-Scioto	
2-Allen		20-Defiance		38-Holmes		56-Monroe		74-Seneca	
3-Ashland		21-Delaware		39-Huron		57-Montgomery		75-Shelby	
4-Ashtabula		22-Erie		40-Jackson		58-Morgan		76-Stark	
5-Athens		23-Fairfield		41-Jefferson		59-Morrow		77-Summit	
6-Auglaize		24-Fayette		42-Knox		60-Muskingum		78-Trumbull	
7-Belmont		25-Franklin		43-Lake		61-Noble		79-Tuscarawas	
8-Brown		26-Fulton		44-Lawrence		62-Ottawa		80-Union	
9-Butler		27-Gallia		45-Licking		63-Paulding		81-Van Wert	
10-Carroll		28-Geauga		46-Logan		64-Perry		82-Vinton	
11-Champaign		29-Greene		47-Lorain		65-Pickaway		83-Warren	
12-Clark		30-Guernsey		48-Lucas		66-Pike		84-Washington	
13-Clermont		31-Hamilton		49-Madison		67-Portage		85-Wayne	
14-Clinton		32-Hancock		50-Mahoning		68-Preble		86-Williams	
15-Columbiana		33-Hardin		51-Marion		69-Putnam		87-Wood	
16-Coshocton		34-Harrison		52-Medina		70-Richland		88-Wyandot	
17-Crawford		35-Henry		53-Meigs		71-Ross		89-Out-of-State	
18-Cuyahoga		36-Highland		54-Mercer		72-Sandusky			

6. **In which of the following sites does your company (branch) treat for bed bugs, and approximately what percentage of your overall bed bug work is done in each? (*Please note that percentages must sum to 100%*.)**

Site	%	Site	%
Hotels/motels		College dormitories	
Apartments/condos--government subsidized		Hospitals/outpatient facilities	
Apartment/condos--non-government subsidized		Doctor's offices	
Single family homes		Commercial/office buildings	
Nursing homes		Vehicles (train/bus/taxi/airplane/etc.)	
Schools/day care centers		Other *(please specify)* ______________________	

7. **Do you have call tracking (<u>software</u>)? *(please circle one)*   Yes   No**

8. **How many total calls did you receive regarding treatment for bed bugs during each of the following years (2010-2016)? Note, if you do not have call tracking, please estimate the number of calls. *(Please check one category for each year.)***

# Calls / Year	0	1-10	11-50	51-100	101-500	501-1000	1001-2000	2001-3000	3001-4000	4001-5000	>5000	Don't Know
**2010**	☐	☐	☐	☐	☐	☐	☐	☐	☐	☐	☐	☐
**2011**	☐	☐	☐	☐	☐	☐	☐	☐	☐	☐	☐	☐
**2012**	☐	☐	☐	☐	☐	☐	☐	☐	☐	☐	☐	☐
**2013**	☐	☐	☐	☐	☐	☐	☐	☐	☐	☐	☐	☐
**2014**	☐	☐	☐	☐	☐	☐	☐	☐	☐	☐	☐	☐
**2015**	☐	☐	☐	☐	☐	☐	☐	☐	☐	☐	☐	☐
**2016**	☐	☐	☐	☐	☐	☐	☐	☐	☐	☐	☐	☐

<u>*Instructions*</u>
Please answer all of the remaining questions based on your company's experience treating bed bugs between 2010 and 2016. For any questions that involve a written response, <u>PLEASE PRINT CLEARLY</u>.

9. **Approximately what percentage of your customers that request bed bug work actually have a pest other than bed bugs (misdiagnosed by customers)?** ______ **%**
   (Please round to nearest whole percent.)

10. **In the table below, please check the box next to all of the insects or things that your customers have misdiagnosed as bed bugs. Then rank all items that you check, starting with 1 being "most frequently misdiagnosed as bed bugs," 2 being "2nd most frequently misdiagnosed as bed bugs," etc.**

**Choices**	**Customers have misdiagnosed as bed bugs? *(Check box if yes.)***	**Rank *(Only rank checked items.)***
Carpet beetles (adults, larvae, or shed skins)	☐	
Cockroaches	☐	
Stink bugs	☐	
Stored product beetles	☐	
Bat bugs	☐	
Non-insect (skin scrapings, lint, debris, etc.)	☐	
Other *(please specify)*:______________________	☐	

11. **In your estimation, what percentage of your customers have attempted do-it-yourself (DIY) treatment(s) for bed bugs?** ***(Please circle one choice from each row.)***

**Before hiring you**	0%	1-10%	11-25%	26-50%	51-75%	>75%
**While you are still conducting the customer's bed bug job**	0%	1-10%	11-25%	26-50%	51-75%	>75%

12. **If you answered a percentage greater than 0% above for either row, please answer parts (a), (b), and (c) below:**

a) How much do you estimate that each of your customers spends, on average per year, for DIY bed bug products/treatments? ***(Please round to the nearest dollar.)*** **$** ______

b) What percent of your customers (who attempt to use DIY products) solve their bed bug infestation using DIY products? ***(Please round to nearest whole percent.)*** ______ **%**

c) If your answer to (b) was greater than 0%, please briefly explain the circumstances of the infestation and the time period that it took to achieve bed bug control.

____

____

13. **In your estimation, what percentage of your customers have misused products in an attempt to kill bed bugs?** ***(Please circle one.)***

0% 1-10% 11-25% 26-50% 51-75% >75% Don't Know

14. **If you are aware of customers misusing products for bed bugs, what products have customers misused?**

**Choices**	**Customers have misused against bed bugs?*(Check box if yes.)***
Alcohol (isopropyl, rubbing, etc.)	☐
Home cleaner/disinfectant (bleach, laundry detergent, etc.)	☐
Petroleum product (gas, oil, etc.)	☐
Over-the-counter aerosol or liquid	☐
Over-the-counter fogger ('bug bomb')	☐
Natural product spray or liquid (botanical, 'green', 12b [non-EPA registered])	☐
Dust/powder pesticide (other than garden dusts) *(please list specific products)* ______________________	☐
Professional pesticide liquid or spray other than garden products (EPA registered; typically not available at retail stores) *(please list specific products)* ______________________	☐
Outdoor/garden pesticide (spray or dust) (*please list specific products*) ______________________	☐
Illegal/unregistered pesticide	☐
Other *(please specify)*:______________________	☐

15. **What percentage of your customers have disposed of their furniture in their efforts to control bed bugs?** ***(please circle one)***

0% 1-10% 11-25% 26-50% 51-75% >75% Don't Know

16. **If you answered a percentage greater than 0% above, to what extent are your customers using the following procedures to dispose of bed bug-infested furniture?** ***(please check one per row)***

	Never	Rarely	Sometimes	Often	Always	Don't Know
Items are wrapped prior to disposal	☐	☐	☐	☐	☐	☐
Items are treated prior to disposal	☐	☐	☐	☐	☐	☐
Prior to disposal, items are ripped, damaged, or otherwise defaced to prevent re-use	☐	☐	☐	☐	☐	☐
Items are placed for normal trash pick-up	☐	☐	☐	☐	☐	☐
Items are taken to the dump by the customer or their designee	☐	☐	☐	☐	☐	☐
Items are given away or sold to someone else	☐	☐	☐	☐	☐	☐

17. **To what extent is your company using the following methods/devices to detect bed bugs? (*please check one per row*)**

	Never	Rarely	Sometimes	Often	Always
Visual inspection	☐	☐	☐	☐	☐
Active monitoring devices (monitors/traps using heat, carbon dioxide [CO_2], chemical lure – either singly or in any combination)	☐	☐	☐	☐	☐
Glueboards or sticky traps	☐	☐	☐	☐	☐
Pitfall traps (typically placed underneath each bed leg)	☐	☐	☐	☐	☐
Canine (dog)-handler team(s)	☐	☐	☐	☐	☐

18. **How many treatments for bed bugs (individual residence, individual unit or apartment treated within an apartment building, company office treated within a larger building, hotel room, etc.) did your company (branch office) conduct during each of the following years (2010-2016)? Note, if you do not have exact numbers, please estimate the number of treatments.** ***(Please check only one box for each year.)***

# Calls / Year	0	1-10	11-50	51-100	101-500	501-1000	1001-2000	2001-3000	>3000	Don't Know	Did not provide treatments
**2010**	☐	☐	☐	☐	☐	☐	☐	☐	☐	☐	☐
**2011**	☐	☐	☐	☐	☐	☐	☐	☐	☐	☐	☐
**2012**	☐	☐	☐	☐	☐	☐	☐	☐	☐	☐	☐
**2013**	☐	☐	☐	☐	☐	☐	☐	☐	☐	☐	☐
**2014**	☐	☐	☐	☐	☐	☐	☐	☐	☐	☐	☐
**2015**	☐	☐	☐	☐	☐	☐	☐	☐	☐	☐	☐
**2016**	☐	☐	☐	☐	☐	☐	☐	☐	☐	☐	☐

19. **On average, how many total visits per infestation are needed for your company to achieve bed bug control?** ***(Please circle one)***

0 1 2 3 4 5 >5

20. **What type of treatments/products do you typically use to control bed bugs?** ***(Check all that apply)***

- ☐ 1-Insecticide/Chemical sprays
- ☐ 2-Insecticide/Chemical dusts
- ☐ 3-Whole room/structure heat treatment
- ☐ 4-Container heat treatment
- ☐ 5-Steam
- ☐ 6-Spot-freezing equipment
- ☐ 7-Structural fumigation (sulfuryl fluoride)
- ☐ 8-Vacuum (other than that performed by customer)
- ☐ 9-Vacuuming performed by customer
- ☐ 10-Disposal of infested items
- ☐ 11-Mattress/box springs encasements
- ☐ 12-Infested items placed inside freezer
- ☐ 13-Laundering of infested items by customer (using washer +/or clothes drier)
- ☐ 14-Insecticide-impregnated liners for mattress/box springs
- ☐ 15-Container fumigation (sulfuryl fluoride)
- ☐ 16-Other *(please specify)*

____

____

21. a) If you checked "insecticide/chemical sprays" above in question 20, what spray treatments/products do you usually use to treat for bed bugs?

- ☐ Bedlam
- ☐ Bedlam Plus
- ☐ Cy-Kick
- ☐ Demand
- ☐ Dragnet
- ☐ Esfenvalerate
- ☐ Gentrol
- ☐ Kicker
- ☐ Onslaught
- ☐ Permanone
- ☐ Phantom
- ☐ Prelude
- ☐ Prentox
- ☐ Saga
- ☐ Steri-Fab
- ☐ Suspend
- ☐ Talstar
- ☐ Tandem
- ☐ Tempo Insecticide
- ☐ Temprid
- ☐ Transport
- ☐ Zenprox
- ☐ CrossFire
- ☐ Other (*please specify*) ______________________

b) If you selected "CrossFire" above in question 21a, please answer the following question: Since this is a newly marketed product, estimate the percentage of jobs in which you have used CrossFire.

☐ **%**

c) If you checked "insecticide/chemical dusts" above in question 20, what dust treatments/products do you usually use to treat for bed bugs?

- ☐ Alpine D
- ☐ CimeXa
- ☐ Cynoff D
- ☐ Delta Dust
- ☐ Drione
- ☐ EcoPCO D/X
- ☐ Perma-Dust
- ☐ Perma-Guard
- ☐ Tempo D
- ☐ Tri-Die
- ☐ Other (*please specify*) ______________________

22. How would you rate your level of satisfaction with the <u>currently available chemical treatment options</u> for bed bugs? *(please circle one)*

1 -Very Dissatisfied	2 - Dissatisfied	3 - Neither Dissatisfied nor Satisfied	4 - Satisfied	5 - Very Satisfied

Please provide comments as to why you are satisfied or dissatisfied with the currently available chemical treatment options.

23. How would you rate your level of satisfaction with the <u>currently available non-chemical treatment options</u> for bed bugs? *(please circle one*)

1 -Very Dissatisfied	2 - Dissatisfied	3 - Neither Dissatisfied nor Satisfied	4 - Satisfied	5 - Very Satisfied

**Please provide comments as to why you are satisfied or dissatisfied with the currently available non-chemical treatment options.**

24. Currently, what percentage of your total treatments fall into the following categories? *(Please note that percentages must sum to 100%.)*

Number of Bed Bugs	Percent of Total Treatments
< 10 bed bugs per infestation (light infestation)	%
11-100 bed bugs per infestation (medium infestation)	%
101-1000 bed bugs per infestation (heavy infestation)	%
>1000 bed bugs per infestation (very heavy infestation)	%

25. a) Among your customers, approximately what percent of multi-family dwellings do not allow inspection of surrounding units? (*If not applicable, please leave blank.)* [ ] **%**

b) Is it your policy to inspect surrounding units (when permitted and possible) within a multi-family dwelling? *(please circle one*)

Yes No Don't know Not applicable

26. Do you feel that your customers are recognizing bed bug infestations earlier now than 6 years ago? *(please circle one*)

Yes No Don't know

27. Do you sell retail items (for bed bugs)? *(please circle one)*

Yes No

28. If you answered Yes to question 27 above, please answer parts (a), (b), and (c) below.
a) What retail products do you offer (for sale or rent)?

☐ Insecticide/chemical spray *If so, how many different spray products?* ________	☐ Glue boards/sticky traps	☐ Steamers
☐ Insecticide/chemical dust *If so, how many different dust products?* ________	☐ Disposal bags/sheeting for furniture or mattress/box springs	☐ Vacuums
☐ Insecticide strips (i.e., Nuvan)	☐ Laundry bags	☐ Inspection kits
☐ Bug bombs	☐ Mattress/box springs encasements	☐ Dust applicators
☐ Bed bug monitors/traps with chemical lure	☐ Mattress/box springs liners	☐ Spray bottles
☐ Pitfall-type traps/interceptors	☐ Portable heat chambers	☐ Other *(please specify)* ____________________

b) Of the retail products that you selected above, what is the one item that <u>customers buy most often</u>? You may write it below or simply circle it from the choices above.

c) Do you provide verbal advice on how to best use these products? *(please circle one)*

Yes No

29. Please provide any additional comments or suggestions regarding bed bugs that you would like to share, or additional clarification of particular answers in this survey.

____

____

____

Page intentionally left blank

Items 30-32 are optional, but known survey participants will be acknowledged, primarily by company name, in presentations and publications outlining the survey results. If you wish to be publicly acknowledged for your help with the survey, please provide the following information. And, THANK YOU for your help with this important endeavor.

30. Your name (optional) ____

31. Your company (optional) ____

32. Your email (optional) ____

Please use the enclosed envelope to return this questionnaire at no cost to you. You may also mail the questionnaire to:

Statistical Consulting Service
The Ohio State University
328 Cockins Hall
1958 Neil Avenue
Columbus OH 43210
