# Supplementary material for: Magnitude and Spread of Bed Bugs (Cimex lectularius) throughout Ohio (USA) Revealed by Surveys of Pest Management Industry
Source: Insects. 2021 Feb 4;12(2):133. doi: 10.3390/insects12020133 (PMC7913827; doi:10.3390/insects12020133)
Supplement: Supplementary file 1 [file insects-12-00133-s001.zip › Exhibit S3--List of Participating Companies.docx]

List of Survey Participants

The following companies participated in one or both (*) of the Ohio Statewide Bed Bug Surveys and elected to provide their name. (This list includes those who responded to the survey, regardless of whether they did bed bug work.) Other participants, not listed, chose to remain anonymous. Appreciation is extended to all survey participants.

- 1 Call Pest Contracting
- 4 Corners Pest Solutions
- 4Sure Pest Control
- *A & A Pest Control Service
- A-ABEL Exterminating Co.
- Abell Pest Control
- ABES Pest Control, Inc.
- Absolute Extermination and Inspection, LLC
- Accel Pest Control OH, LLC
- *Ace Exterminating Co.
- Acme Exterminating, Inc.
- Action Exterminating
- Action Now, Inc.
- Action Pest Control, Inc. & American Exterminating Co.
- Advanced Pest Control
- Aero Pest Control, LLC
- Affordable Exterminating
- *Aid Pest Control, LLC
- Algo Termite and Pest Control
- All Ohio Pest Control Co.
- All Pest Exterminating, Inc.
- AllPest Pest Control and Solutions
- All State Termite & Pest
- All-Pro Wildlife Control
- Alright Pest Solutions LLC
- Alron Pest Control
- Ampulex Environmental Solutions, LLC
- Andy's Alliance Pest Control
- Antrim Pest Control
- *Apollo Pest Control
- *Arab Termite and Pest Control
- Arnold-Wilmar Pest Solutions
- Arrest-A-Pest of Northern Ohio
- Avalon Pest Control
- Ayers Pest Mgt., LLC
- Bay Pest Control
- BCG, LLC
- Beastmaster Pest Services
- Bed Bug Burners, LLC
- Bob Seljan
- Boggs Pest Control, Inc.
- *Bona Fide Commercial Services
- Brookside Lawn Service, Inc.
- Bryan Hartley
- Buckeye Bug Blasters, LLC
- *Buckeye Exterminating, Inc.
- Budget Pest Control
- Buentello Pest Control
- Bug Off Pest Control
- Bug Zappers
- *Capital City Exterminating Co.
- Carothers Pest Control
- Cavalry Pest Control
- CCI Wireless
- Central Exterminating Co., Inc.
- Central Ohio Exterminating
- Certified Extermination Solution
- Christensen's Urban Insect Solutions, Inc.
- Classic Care
- *Cleveland Chemical Pest Control, Inc.
- Clippinger Pest Control
- *Columbus Pest Control, Inc.
- Complete Termite & Pest Control
- Contact Pest Control, LLC
- Couch's Termite & Pest Control
- Crankerup Pest Control
- Critter Control of Cincinnati
- Critter Control of Lexington
- Cuyahoga Metro. Housing Authority
- D/J Bug Away, LLC
- Dan's Pest Patrol
- David Hersh
- Dayton's Bed Bug Dog, LLC
- Dependable Pest Control
- Dick's All Be Gone Pest Control Ltd.
- Discreet Bed Bug Removal
- *Discreet Pest Control
- Eco-Care Pest Control
- Economy Pest Control
- Ecopro Pest Solutions
- Elite Pest Management, LLC
- *Epcon Lane
- Extermital Termite & Pest Control, Inc.
- Faith Enterprise Environmental Services
- First Choice Pest Control
- First Choice Pest Management Co.
- Flag City Pest Control, Inc.
- Frame's Pest Control, Inc.
- Friend's Termite & Pest Control
- Frobase Horticultural Services
- Gary Benson Pest Control
- Gary L. Bauer
- Geauga Metro. Housing Authority
- Gotcha! Pest Control Specialists, Inc.
- Grass Master, Inc.
- Great Lakes Pest Management Services
- Hartley's Termite & Pest Control, LLC
- Hawx Pest Control
- Hixco Integrated Pest Management
- Holmes Pest Control, Inc.
- Homewood Pest Control, LLC
- *Hooper Termite & Pest Control
- House Physicians of Ohio
- Imperial Pest Control, LLC
- Innovative Pest Management, Inc.
- InspectOhio
- *Integrity Pest Solutions, LLC
- JB Vegetation & Insect Control, LLC
- John Henry's Pest Control
- Joy Exterminating Co., LLC
- Keith's Perfect Fit, LLC
- Kline Pest Control Co., Inc.
- L&S Termite and Pest Control, Inc.
- Ladybug Services, LLC
- Lakewood Exterminating
- Lawnco, Inc.
- Licking Metro. Housing Authority
- Liechty & Sons Exterminating
- LongPro Pest Control
- Lucky Lee's Pest Control
- Lu-Crest Pest Control
- Lutes Flying Service, Inc.
- Mauger Exterminating Co.
- *Meredith Pest Control
- Merlin's Pest Control
- Miami University
- Miami Valley Pest Control Service
- *Michael’s Pest Control
- Midwest Termite & Pest Control
- Moore Pest Management Co.
- Mulholland Pest Control, Inc.
- *Ohio Exterminating Co., Inc.
- Ohio Valley Pest Control
- *Orkin, LLC (multiple branches)
- Paragon Pest Elimination
- Patrick's Pest Control
- Pawnee Pest Management
- PCS Lawncare
- Pepzee Realty
- *Permakil Pest Control, Inc.
- Pesco Pest Control, LLC
- Pest Pro Pest Solutions, Inc.
- *Pest-All Exterminating
- Phelps Termite & Pest Control
- Pike Professional Pest Control
- *Precision Pest Management
- Premier Pest Control, LLC
- Prevent Pest Control
- *Pro Kill, Inc.
- Prokill Exterminating
- Redwine Pest Control
- Reliance Home Inspections
- Responsible Services
- Rid-X Pest Control, Inc.
- *Rose Pest Solutions
- RxProtect
- S.A.B. Landscaping, Inc.
- Scherzinger Termite & Pest Control Management, LLC
- Scioto County Career Tech
- Seckman Pest Control
- Sideline Property
- *Skyhigh Termite & Pest Control, LLC
- Snowball Pest Control
- *Speed Exterminating Co., Inc.
- SPS Pest Control, Inc.
- State Termite & Pest Solutions
- Stewart Pest Control Co.
- Stauffs Corp. DBA: Aid Pest Control
- Sure Thing Pest Control
- T&M Pest Control, Inc.
- Terminator Services
- Terminix (multiple branches)
- Termitco
- *Terry the Bug Man
- The Bug Guy Tom
- The X-Terminator
- TNT Exterminating Co.
- Tom's Pest Control
- Tony Shultz
- Torco
- TriCity Termite
- Truly Nolen, Inc. (multiple branches)
- Ultra Pest Control
- Universal Pest Control
- Valley Termite & Pest Control, LLC
- Vandagriff Pest Control
- Varment Guard
- Vaughn Pest Control, LLC
- Vegetation Solutions
- Vollman Pest Control
- Wells Thur-O Pest Control, Inc.
- William A Barns Trust
- Wright's Termite Pest Co.
- Yards Done Right
- Ziehler Lawn and Tree Care, LLC
